# Examining the relationships between mindfulness, emotion regulation, depression, and anxiety: a structural equation modeling approach

**DOI:** 10.3389/fpsyg.2026.1738432

**Published:** 2026-03-02

**Authors:** Hanan Alfayez

**Affiliations:** Department of Psychology, King Saud University, Riyadh, Saudi Arabia

**Keywords:** anxiety, cognitive reappraisal, depression, emotion regulation, expressive suppression, mindfulness, Saudi Arabia

## Abstract

**Introduction:**

This study examined the pathways linking mindfulness to emotion regulation and psychological symptoms among adults in Saudi Arabia.

**Methods:**

A total of 306 adults completed validated measures of mindfulness, emotion regulation (cognitive reappraisal and expressive suppression), depression, and anxiety. Structural equation modeling was used to test a hypothesized model in which emotion regulation mediated the relationship between mindfulness and psychological wellbeing.

**Results:**

Mindfulness was positively associated with cognitive reappraisal but unrelated to expressive suppression. Cognitive reappraisal predicted lower levels of depression and anxiety, whereas expressive suppression was associated with higher psychological distress. Unexpectedly, mindfulness demonstrated small positive direct associations with depression and anxiety once emotion regulation variables were included, suggesting partial suppression effects. Mediation analyses indicated that emotion regulation partially mediated the mindfulness-symptom relationship, mediation, though total effects were nonsignificant.

**Discussion:**

The findings highlight the dual role of mindfulness, which may enhance emotional awareness while simultaneously increasing sensitivity to distress when regulation skills are limited. Culturally, the results emphasize the need to adapt mindfulness-based interventions to align with Saudi values and traditions. Future research should employ longitudinal designs and explore culturally grounded forms of mindfulness practice to better understand these.

## Introduction

Mental health challenges such as depression and anxiety represent some of the most pressing public health concerns worldwide, with growing prevalence rates across both clinical and non-clinical populations (World Health Organization, 2023). In the Middle East, and particularly within Saudi Arabia, the rapid pace of social change, economic transitions, and shifting cultural dynamics have placed additional psychological demands on individuals, intensifying the need for effective strategies that enhance resilience and psychological wellbeing (Al-Hanawi et al., 2020). Against this backdrop, the study of mindfulness and its association with emotional regulation has emerged as a promising avenue for understanding how individuals manage distress and maintain mental health stability.

Mindfulness is commonly defined as the intentional and non-judgmental awareness of the present moment (Kabat-Zinn, 2003). Conceptually, it can be understood both as a state - a temporary condition cultivated through practice - and as a trait, referring to a relatively stable individual disposition toward being attentive and aware of present experiences (Brown and Ryan, 2003; Baer et al., 2006). While mindfulness as a state reflects moment-to-moment awareness induced by meditation or attention-based exercises, dispositional mindfulness represents a more enduring tendency that influences how individuals habitually perceive and respond to emotional stimuli. This distinction is central to the present study, which focuses on trait mindfulness as an individual characteristic associated with emotion regulation and mental health outcome. Research has consistently demonstrated that higher levels of trait mindfulness are linked to lower symptoms of depression and anxiety, likely through enhanced regulatory processes and reduced maladaptive cognitive patterns such as rumination (Kuyken et al., 2010; Hadjiilieva, 2025).

Emotion regulation refers to the processes - cognitive, behavioral, and physiological-through which individuals influence the intensity, duration, and expression of their emotions (Gross, 2002; Bridges et al., 2004). Among the strategies most frequently studied are cognitive reappraisal, which involves reframing the meaning of an emotional stimulus, and expressive suppression, which involves inhibiting outward signs of emotion. Theoretical and empirical evidence suggests that these strategies have differential consequences: cognitive reappraisal is generally associated with improved wellbeing and lower psychological distress, while suppression is linked to heightened depressive and anxious symptoms (Campbell-Sills and Barlow, 2006; Desrosiers et al., 2013; Peh et al., 2017).

From a theoretical standpoint, Gross’s (2002) process model of emotion regulation provides a compelling framework for understanding how mindfulness shapes emotional functioning. Within this model, emotions unfold through a sequence of stages, ranging from situation selection and attentional deployment to cognitive change and response modulation, each offering an opportunity for regulatory influence. Mindfulness, characterized by purposeful and nonjudgmental awareness of present-moment experience, may enhance adaptive regulation by increasing sensitivity to early emotional cues and internal states. This assertion is supported by empirical findings suggesting that mindfulness promotes interoceptive awareness and attentional control, skills that enable individuals to detect emotional activation earlier and apply reappraisal strategies more effectively (Teper et al., 2013; Lindsay and Creswell, 2017). This heightened awareness facilitates the timely use of constructive strategies such as cognitive reappraisal, allowing individuals to reinterpret emotionally charged situations in a more balanced manner. In parallel, mindfulness reduces reliance on maladaptive strategies such as suppression, which often exacerbate physiological arousal and hinder emotional processing (Keng et al., 2013; Garland et al., 2015b). These mechanisms justify the assumption that mindfulness contributes to adaptive emotional functioning through attentional refinement, metacognitive monitoring, and acceptance-based processing.

Recent integrative frameworks have expanded on Gross’s model, proposing that mindfulness fosters a meta-awareness that allows individuals to observe emotional experiences from a decentered perspective rather than being overwhelmed by them (Garland et al., 2015b; Brockman et al., 2017). This metacognitive stance promotes openness and acceptance, enabling individuals to disengage from automatic or habitual emotional reactions and to respond with greater flexibility (Parmentier et al., 2019). Empirical findings consistently support this theoretical link: mindfulness training has been associated with enhanced cognitive reappraisal and reduced expressive suppression, indicating a shift toward more adaptive regulatory patterns (Zhou et al., 2023; Pang et al., 2025). Moreover, studies employing structural equation modeling have shown that emotion regulation mediates the associations between mindfulness and psychological symptoms, suggesting that the interplay among these constructs can be quantitatively modeled to capture their convergent validity and directional relationships (Desrosiers et al., 2013; Garland et al., 2015a; Freudenthaler et al., 2017).

Despite growing international evidence, research exploring these mechanisms in Arab societies remains limited. Cultural norms regarding emotional expression, coping, and mental health stigma may influence how mindfulness and emotion regulation are enacted and experienced in non-Western settings (Dwairy, 2006). For example, in collectivist cultures, suppression may sometimes be socially adaptive, yet it can still contribute to long-term psychological strain. Given these sociocultural nuances, there is a clear need to evaluate mindfulness and its correlates in the Saudi population, not only to test the cross-cultural generalizability of existing models but also to inform culturally adapted interventions that enhance emotional wellbeing and resilience. Such exploration holds both theoretical and applied value, as it can clarify whether established Western frameworks of mindfulness and emotion regulation accurately capture emotional functioning in Arab contexts.

Building on this foundation, the present study seeks to clarify the structural relationships between mindfulness, emotion regulation strategies (cognitive reappraisal and expressive suppression), and psychological symptoms of depression and anxiety. Specifically, the study examines (a) the pathways linking mindfulness to emotion regulation strategies, (b) the associations between emotion regulation strategies and symptoms of depression and anxiety, and (c) whether mindfulness exerts a direct negative influence on distress. In addition, it explores whether emotion regulation mediates the relationship between mindfulness and psychological symptoms, thereby contributing to a more comprehensive understanding of their interrelations within a non-Western cultural framework.

## Methods

### Participants

The study sample consisted of 306 adults residing in Saudi Arabia. Participants were recruited from the general population through online invitations distributed via email and social media platforms. The recruitment targeted individuals aged 18 years and above, regardless of gender or occupational background. Participation was voluntary, and no financial compensation was provided. Before participation, all individuals were informed about the purpose of the study, confidentiality of data, and their right to withdraw at any time without consequence. Only those who provided informed consent were included in the study. Inclusion criteria were: (a) age 18 years or older, (b) ability to read and understand Arabic, and (c) current residency in Saudi Arabia. Exclusion criteria included: (a) inability to provide informed consent.

### Measures

#### Mindfulness

Mindfulness was assessed using the Five Facet Mindfulness Questionnaire (FFMQ) (Baer et al., 2006), which comprises 39 items covering five components: observing, describing, acting with awareness, nonjudging of inner experience, and nonreactivity to inner experience. Participants rated each statement on a 5-point Likert scale ranging from 1 (“never or very rarely true”) to 5 (“very often or always true”). Scores for negatively worded items were reverse coded prior to analysis. Subscale scores were computed by summing responses for each facet, and the total mindfulness score was obtained by summing across all items, with higher scores reflecting greater dispositional mindfulness. The Arabic version of the FFMQ has been employed and evaluated in Arabic-speaking samples, including studies that sampled participants from Saudi Arabia, supporting its cross-cultural applicability (Albuhairi et al., 2021). In the present sample, the Arabic version demonstrated satisfactory composite reliability for all subscales: Observing (CR = 0.843), Describing (CR = 0.822), Acting with Awareness (CR = 0.881), Nonjudging (CR = 0.864), and Nonreactivity (CR = 0.740), supporting the internal consistency of the measure.

#### Emotion regulation

Emotion regulation was measured using the Emotion Regulation Questionnaire (ERQ) (Gross and John, 2003). The ERQ includes 10 items divided into two subscales: cognitive reappraisal (6 items) and expressive suppression (4 items). Each item was rated on a 7-point Likert scale ranging from 1 (“strongly disagree”) to 7 (“strongly agree”). Mean subscale scores were calculated, with higher reappraisal scores indicating greater use of adaptive emotion regulation strategies, and higher suppression scores indicating greater reliance on maladaptive strategies. The ERQ has validated Arabic translations reported in the literature and has been used in regional samples (Merhi and Kazarian, 2015). In this study, the Arabic version of the ERQ demonstrated good composite reliability for both subscales: Cognitive Reappraisal (CR = 0.824) and Expressive Suppression (CR = 0.804).

#### Depressive symptoms

Depressive symptoms were assessed using the Patient Health Questionnaire-9 (PHQ-9) (Kroenke et al., 2001), a self-report measure based on DSM-5 criteria for depression. Participants rated how often they experienced each of nine symptoms during the previous 2 weeks on a 4-point scale (0 = “not at all” to 3 = “nearly every day”). The total PHQ-9 score ranges from 0 to 27, with higher scores indicating more severe depressive symptoms. Severity levels are typically classified as minimal (0–4), mild (5–9), moderate (10–14), moderately severe (15–19), and severe (20–27). The Arabic version of the PHQ-9 has been previously validated and widely used in studies conducted in Saudi Arabia, demonstrating excellent psychometric properties and cultural applicability (Alhadi et al., 2017). In the present study, the PHQ-9 showed excellent composite reliability in this sample (CR = 0.957), indicating strong internal consistency.

#### Anxiety symptoms

Anxiety was measured using the Generalized Anxiety Disorder Scale (GAD-7) (Spitzer et al., 2006), which includes seven items assessing the frequency of anxiety-related symptoms during the previous 2 weeks. Responses are rated on a 4-point scale (0 = “not at all” to 3 = “nearly every day”). The total score ranges from 0 to 21, with higher scores reflecting greater anxiety severity. Established clinical cutoffs categorize anxiety as minimal (0–4), mild (5–9), moderate (10–14), or severe (15–21). The Arabic GAD-7 has been validated in multiple Arabic-language studies and demonstrates acceptable psychometric properties (Khoury-Malhame et al., 2025). In the current study, the Arabic version of the GAD-7 demonstrated excellent composite reliability (CR = 0.929), supporting its robust internal consistency.

### Procedure

Data collection was conducted online over a three-month period between July and September 2025. The survey link was distributed via institutional email lists, WhatsApp groups, and popular social media platforms such as X (formerly Twitter) and Instagram to reach a diverse audience. Participants accessed the questionnaire via a secure online form that included the informed consent statement and all study measures in Arabic. Participation was voluntary, and respondents could withdraw at any time before submitting their answers. After providing consent, all items were required to ensure complete responses for statistical analyses, which is a common practice in online survey-based research. The survey took approximately 15 min to complete. All responses were reviewed for completeness and consistency before inclusion in the final dataset. Ethical approval for the study was granted by The Humanities College Research (Research Ethics Committee) at King Saud University (Ref no. KSU-HE-25-233).

### Statistical analysis

All statistical analyses were conducted using R (version 4.5.1; R Foundation for Statistical Computing, Vienna, Austria). Prior to testing the hypothesized relationships, a confirmatory factor analysis (CFA) was performed to examine the adequacy of the measurement model, which included the Five Facet Mindfulness Questionnaire (FFMQ), the Emotion Regulation Questionnaire (ERQ), and the measures of anxiety and depression (GAD and PHQ, respectively).

All negatively worded FFMQ items were reverse-scored according to the scoring manual before the analyses. Preliminary data screening was conducted to verify data accuracy, missing values, and distributional assumptions. Normality was assessed through visual inspection of histograms and formal tests of normality (Shapiro–Wilk). As expected, given the sample size, the Shapiro–Wilk tests were statistically significant; however, visual inspection of the distributions indicated no severe departures from normality. Given the robustness of estimation methods commonly used in structural equation modeling (SEM) to moderate deviations from normality, the analyses were deemed appropriate.

The CFA and subsequent structural equation modeling (SEM) were estimated using robust methods to account for the characteristics of the data. Model fit was evaluated using multiple indices: Comparative Fit Index (CFI), Tucker–Lewis Index (TLI), the Root Mean Square Error of Approximation (RMSEA) with its 90% confidence interval, and the Standardized Root Mean Square Residual (SRMR). Consistent with commonly accepted criteria, CFI and TLI values of 0.90 or higher were considered indicative of acceptable model fit, while RMSEA and SRMR values of 0.08 or lower were interpreted as reflecting adequate model fit (Hu and Bentler, 1999; Kline, 2023).

Composite reliability (CR) was used to assess internal consistency instead of Cronbach’s alpha, as CR provides a more accurate estimate within the structural equation modeling framework. Unlike Cronbach’s alpha, which assumes equal indicator loadings, CR accounts for the actual factor loadings derived from the confirmatory factor analysis, thus offering a more precise measure of construct reliability (Raykov, 1997; Hair et al., 2019).

After confirming the adequacy of the measurement model, SEM was conducted to test the proposed structural paths among mindfulness, emotion regulation, anxiety, and depression. All statistical tests were two-tailed, and statistical significance was set at *p* < 0.05.

## Results

### Demographic and clinical characteristics

A total of 306 participants were included in the analysis. The majority were young adults aged 18–34 years (65.7%), and females comprised 69.6% of the sample. Most respondents were Saudi nationals (91.5%), single (52.6%), and held a bachelor’s degree (60.1%). In terms of occupation, the largest subgroup consisted of employees or freelancers (37.9%), followed by students (28.8%). Economically, most participants reported a middle-income level (65.4%).

Regarding mental health history, 82.7% had never been diagnosed with a psychiatric condition. Among those who reported a diagnosis, the most common were anxiety (60.8%) and depression (47.1%), followed by less frequent conditions such as ADHD (5.9%) and PTSD (3.9%).

Concerning mindfulness-related practices, 54.2% indicated that they did not engage in meditation or mindfulness, whereas 45.8% reported doing so regularly. Among practitioners, the most frequent practice schedule was two to three times per week (38.6%), followed by once weekly (34.3%). Full demographic and clinical details are summarized in Table 1.

**Table 1:** Demographic and clinical characteristics.

Characteristic	Category	*n* (%)
Age (years)	18–34	201 (65.7%)
	35–54	78 (25.5%)
	≥ 55	27 (8.8%)
Gender	Male	93 (30.4%)
	Female	213 (69.6%)
Nationality	Saudi	280 (91.5%)
	Non-Saudi	26 (8.5%)
Marital status	Single	161 (52.6%)
	Married	120 (39.2%)
	Divorced	21 (6.9%)
	Widowed	4 (1.3%)
Educational level	Secondary school or less	30 (9.8%)
	Bachelor	184 (60.1%)
	Postgraduate	92 (30.1%)
Occupational level	Not student/ Unemployed	66 (21.6%)
	Employee/Freelance	116 (37.9%)
	Student	88 (28.8%)
	Student and Employee	36 (11.8%)
Economic level	Below middle	29 (9.5%)
	Middle	200 (65.4%)
	Above middle	77 (25.2%)
Ever diagnosed with a psychiatric illness	No	253 (82.7%)
	Yes	53 (17.3%)
If yes, please mention	Anxiety	31 (60.8%)
	Depression	24 (47.1%)
	ADHD	3 (5.9%)
	PTSD	2 (3.9%)
	Stress	2 (3.9%)
	Bipolar disorder	1 (2.0%)
	OCD	1 (2.0%)
	Postpartum depression	1 (2.0%)
	Psychosis	1 (2.0%)
	Social anxiety	1 (2.0%)
Practice meditation or mindfulness	No	166 (54.2%)
	Yes	140 (45.8%)
If yes, the frequency of meditation/mindfulness per week	Once	48 (34.3%)
	2 to 3 times	54 (38.6%)
	More than 3 times	35 (25.0%)

**Figure 1:**
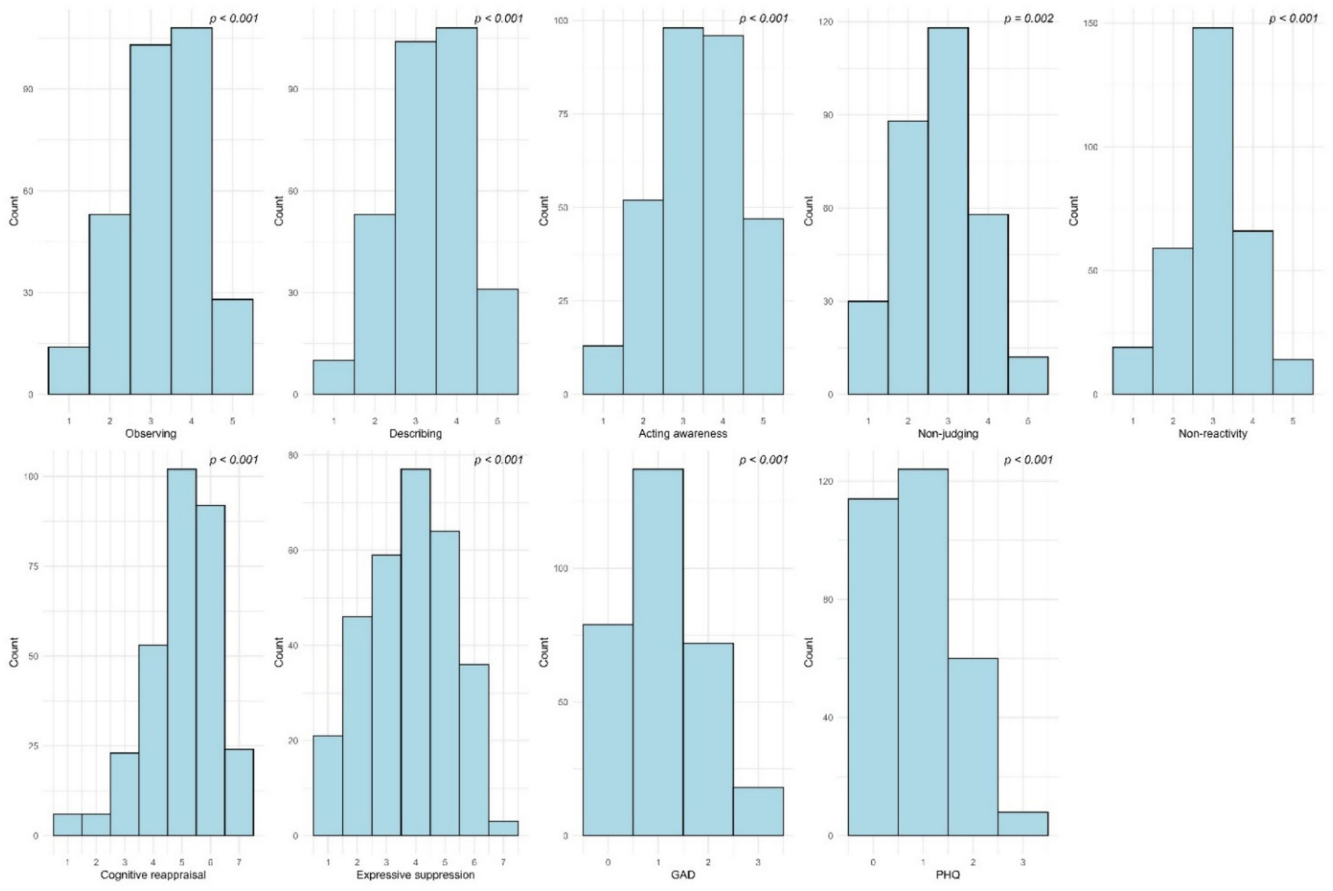
Distributions of composite scores across study variables. Histograms illustrate the observed distributions for mindfulness (five FFMQ facets), emotion regulation (ERQ reappraisal and suppression), anxiety (GAD), and depression (PHQ) measures.

Descriptive statistics and distributional characteristics are shown in Figure 1. Although formal tests indicated deviations from normality, visual inspection suggested no severe departures. Given the use of robust estimation methods within the SEM framework, which are resilient to moderate non-normality, no additional data transformations or alternative analytical procedures were required.

### Results of the measurement model

The hierarchical confirmatory factor analysis (CFA) showed an overall acceptable model fit, indicating that the data adequately represented the proposed latent structure (*N =* 306, 146 parameters). Model fit indices were within an acceptable range—*χ*^2^(1684) = 3530.999, *p* < 0.001; RMSEA = 0.060 (90% CI [0.057, 0.063]); CFI = 0.81; TLI = 0.80; SRMR = 0.16—consistent with complex hierarchical models.

**Figure 2:**
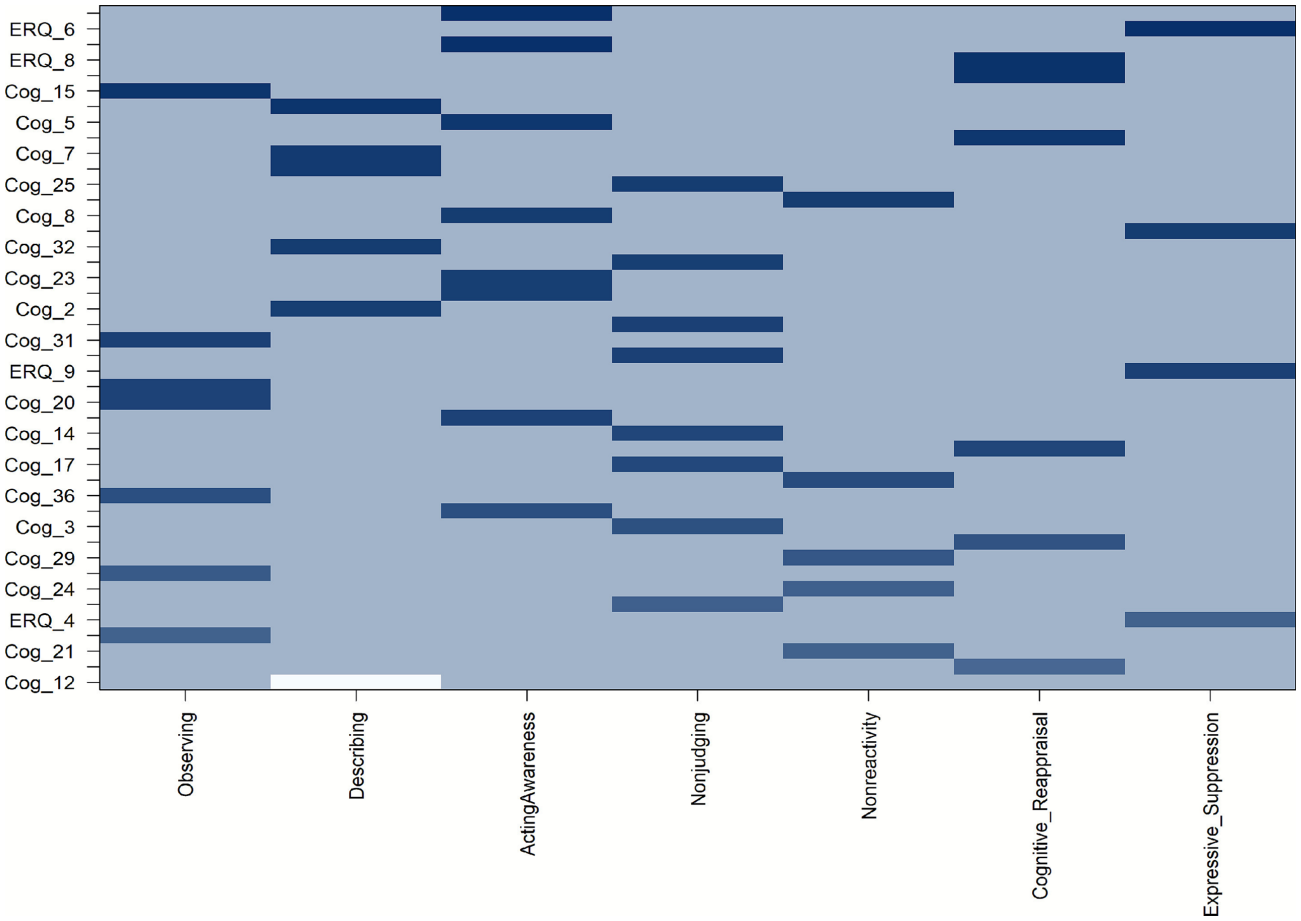
Heatmap of standardized factor loadings across latent constructs. Darker shades indicate stronger item–factor associations; lighter shades indicate negligible cross-loadings.

All indicators loaded significantly on their respective latent constructs, with standardized loadings ranging from moderate to high (Table 2). The mindfulness facets demonstrated robust loadings across all subscales: Observing (0.53–0.81), Describing (0.71–0.86), Acting with Awareness (0.64–0.82), Nonjudging (0.56–0.73), and Nonreactivity (0.51–0.73). Emotion regulation indicators also performed adequately—Cognitive Reappraisal (0.46–0.81) and Expressive Suppression (0.52–0.82). Clinical outcomes (GAD and PHQ) displayed consistently strong loadings (0.54–0.82), confirming the reliability of these constructs. To facilitate interpretation of the factorial structure given the large number of observed indicators, Figure 2 presents a heatmap visualization of standardized factor loadings, highlighting the relative strength of item–factor associations and the absence of substantial cross-loadings across latent constructs. Standardized factor loadings with confidence intervals are reported in detail in Table 2.

**Table 2:** Standardized factor loadings (95% CI) for indicators on their latent constructs from the finalized CFA model.

Variable	Construct	Factor loading (95% CI)
Cog_1	Observing	0.53 (0.44–0.62)
Cog_6	Observing	0.58 (0.50–0.66)
Cog_15	Observing	0.81 (0.76–0.86)
Cog_20	Observing	0.70 (0.64–0.77)
Cog_26	Observing	0.67 (0.60–0.74)
Cog_31	Observing	0.67 (0.60–0.75)
Cog_36	Observing	0.61 (0.54–0.69)
Cog_2	Describing	0.73 (0.67–0.79)
Cog_7	Describing	0.76 (0.70–0.82)
Cog_12	Describing	0.86 (0.69–1.03)
Cog_27	Describing	0.77 (0.72–0.83)
Cog_32	Describing	0.71 (0.64–0.77)
Cog_37	Describing	0.75 (0.69–0.81)
Cog_5	ActingAwareness	0.75 (0.69–0.81)
Cog_8	ActingAwareness	0.72 (0.66–0.78)
Cog_13	ActingAwareness	0.81 (0.76–0.86)
Cog_18	ActingAwareness	0.70 (0.64–0.77)
Cog_23	ActingAwareness	0.72 (0.66–0.78)
Cog_28	ActingAwareness	0.64 (0.56–0.71)
Cog_34	ActingAwareness	0.70 (0.63–0.76)
Cog_38	ActingAwareness	0.82 (0.78–0.87)
Cog_3	Nonjudging	0.63 (0.55–0.71)
Cog_10	Nonjudging	0.68 (0.61–0.75)
Cog_14	Nonjudging	0.67 (0.60–0.74)
Cog_17	Nonjudging	0.68 (0.61–0.75)
Cog_25	Nonjudging	0.72 (0.66–0.79)
Cog_30	Nonjudging	0.73 (0.67–0.80)
Cog_35	Nonjudging	0.56 (0.48–0.65)
Cog_39	Nonjudging	0.72 (0.65–0.78)
Cog_19	Nonreactivity	0.64 (0.56–0.73)
Cog_21	Nonreactivity	0.51 (0.41–0.60)
Cog_24	Nonreactivity	0.54 (0.44–0.63)
Cog_29	Nonreactivity	0.57 (0.48–0.66)
Cog_33	Nonreactivity	0.73 (0.66–0.81)
ERQ_1	Cognitive reappraisal	0.46 (0.37–0.56)
ERQ_3	Cognitive reappraisal	0.60 (0.52–0.68)
ERQ_5	Cognitive reappraisal	0.67 (0.60–0.74)
ERQ_7	Cognitive reappraisal	0.79 (0.73–0.84)
ERQ_8	Cognitive reappraisal	0.81 (0.76–0.86)
ERQ_10	Cognitive reappraisal	0.80 (0.75–0.85)
ERQ_2	Expressive suppression	0.73 (0.66–0.80)
ERQ_4	Expressive suppression	0.52 (0.43–0.62)
ERQ_6	Expressive suppression	0.82 (0.76–0.88)
ERQ_9	Expressive suppression	0.71 (0.63–0.78)
GAD_1	GAD	0.75 (0.70–0.81)
GAD_2	GAD	0.79 (0.74–0.84)
GAD_3	GAD	0.82 (0.77–0.86)
GAD_4	GAD	0.79 (0.74–0.84)
GAD_5	GAD	0.73 (0.67–0.79)
GAD_6	GAD	0.75 (0.69–0.80)
GAD_7	GAD	0.70 (0.64–0.76)
PHQ_1	PHQ	0.69 (0.63–0.76)
PHQ_2	PHQ	0.78 (0.73–0.83)
PHQ_3	PHQ	0.81 (0.77–0.85)
PHQ_4	PHQ	0.76 (0.70–0.81)
PHQ_5	PHQ	0.76 (0.71–0.81)
PHQ_6	PHQ	0.78 (0.74–0.83)
PHQ_7	PHQ	0.75 (0.69–0.80)
PHQ_8	PHQ	0.64 (0.56–0.71)
PHQ_9	PHQ	0.54 (0.45–0.62)

Composite reliability (CR) coefficients indicated strong internal consistency across most domains, generally exceeding recommended thresholds (≥ 0.70). The highest reliability values were observed for PHQ (0.96) and GAD (0.93), followed by Acting with Awareness (0.88), Nonjudging (0.86), and Observing (0.84). The average variance extracted (AVE) values demonstrated satisfactory convergent validity for most constructs, with several domains approaching the recommended criterion (Table 3).

**Table 3:** Composite reliability (CR) and average variance extracted (AVE) for each latent domain.

Domain	Reliability	AVE
Observing	0.843	0.443
Describing	0.82	0.589
Acting Awareness	0.881	0.538
Nonjudging	0.864	0.459
Nonreactivity	0.740	0.361
Cognitive reappraisal	0.824	0.455
Expressive suppression	0.804	0.514
GAD	0.929	0.593
PHQ	0.957	0.560

### Results of the structural model

The structural equation model demonstrated coherent relationships among mindfulness, emotion regulation, and psychological outcomes (Figure 2). Higher mindfulness significantly predicted greater cognitive reappraisal (*β* = 0.439, 95% CI [0.197–0.682], *p* < 0.001) but was unrelated to expressive suppression (*β* = −0.128, 95% CI [−0.449–0.194], *p* = 0.437).

Cognitive reappraisal was associated with lower levels of anxiety (GAD: *β* = −0.148, *p* = 0.005) and depression (PHQ: *β* = −0.172, *p* < 0.001), whereas expressive suppression was related to higher anxiety (*β* = 0.182, *p* < 0.001) and depression (*β* = 0.178, *p* < 0.001).

**Figure 3:**
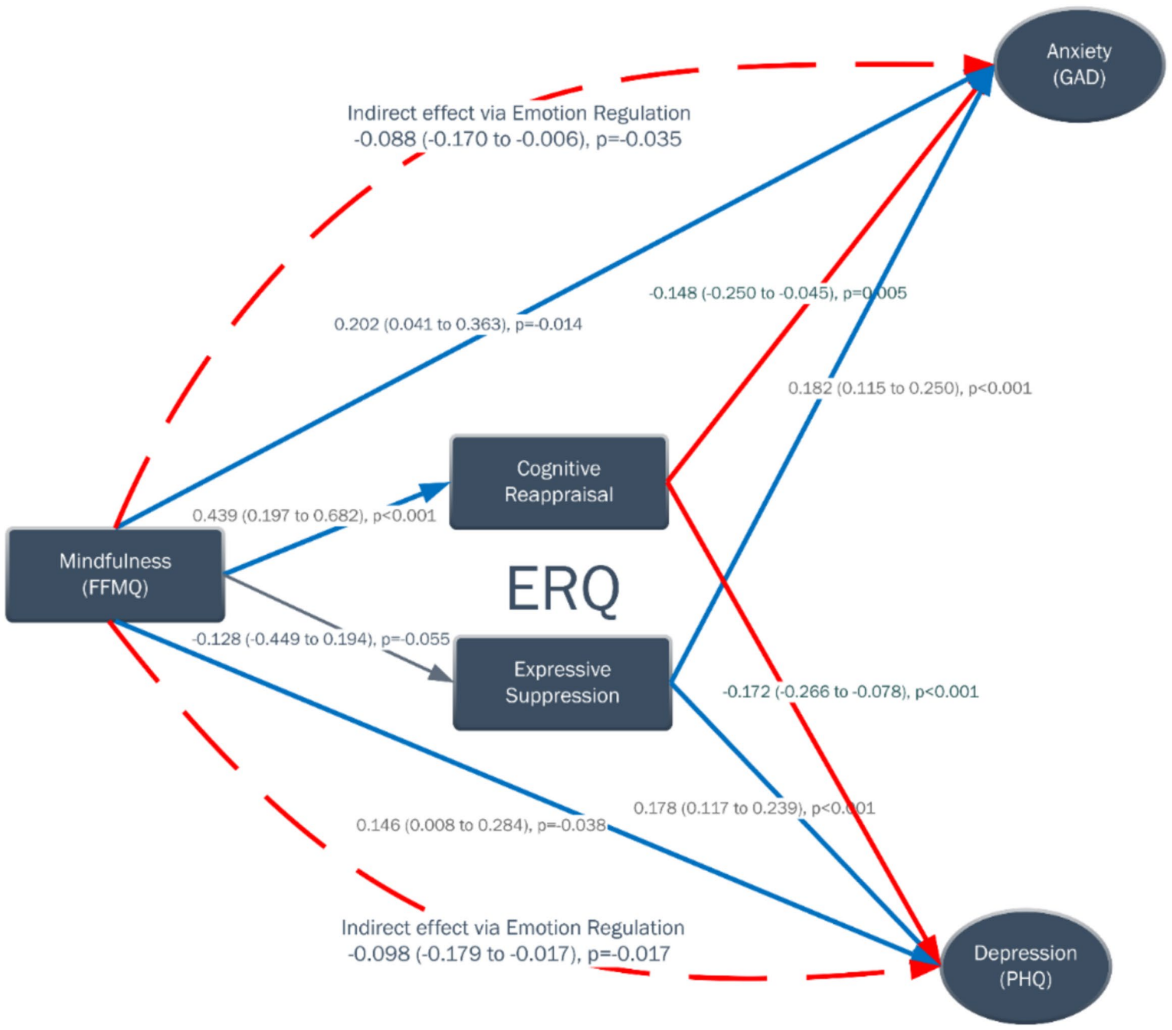
Latent-level representation of the measurement and structural model, illustrating the relationships among mindfulness facets, emotion regulation.

Interestingly, mindfulness also showed small but significant direct paths to both anxiety (*β* = 0.202, *p* = 0.014) and depression (*β* = 0.146, *p* = 0.038), suggesting potential suppression effects when emotion regulation is included in the model (Table 4; Figure 3).

**Table 4:** Structural paths among latent variables.

Path	Beta (95% CI)	*p*-value	Standardized beta
Mindfulness → Cognitive reappraisal	0.439 (0.197 to 0.682)	<0.001	0.288
Mindfulness → Expressive suppression	−0.128 (−0.449 to 0.194)	0.437	−0.055
Cognitive reappraisal → GAD	−0.148 (−0.25 to −0.045)	0.005	−0.193
Expressive suppression → GAD	0.182 (0.115 to 0.25)	<0.001	0.362
Cognitive reappraisal → PHQ	−0.172 (−0.266 to −0.078)	<0.001	−0.250
Expressive suppression → PHQ	0.178 (0.117 to 0.239)	<0.001	0.393
Mindfulness → GAD	0.202 (0.041 to 0.363)	0.014	0.173
Mindfulness → PHQ	0.146 (0.008 to 0.284)	0.038	0.140

### Mediation analysis

As summarized in Table 5, mindfulness exerted small but statistically significant indirect effects on both anxiety and depression through emotion-regulation mechanisms (GAD: *β* = −0.088, 95% CI [−0.170, −0.006], *p* = 0.035; PHQ: *β* = −0.098, 95% CI [−0.179, −0.017], *p* = 0.017).

**Table 5:** Mediation results: indirect and total effects of mindfulness on anxiety and depression via cognitive reappraisal and expressive suppression.

Path	Beta (95%CI)	*p*-value	Standardized beta
Indirect GAD	−0.088 (−0.17 to −0.006)	0.035	−0.076
Total GAD	0.114 (−0.042 to 0.27)	0.153	0.098
Indirect PHQ	−0.098 (−0.179 to −0.017)	0.017	−0.094
Total PHQ	0.048 (−0.089 to 0.185)	0.491	0.046

However, total effects—combining direct and indirect components—were non-significant for both outcomes (GAD: *β* = 0.114, *p* = 0.153; PHQ: *β* = 0.048, *p* = 0.491), indicating that the negative mediated pathways are counterbalanced by small positive direct associations.

These results suggest that while mindfulness enhances adaptive emotion regulation strategies that mitigate anxiety and depression, its overall influence on psychological distress may operate through multiple, partially opposing mechanisms.

## Discussion

The present study aimed to examine the pathways linking mindfulness to emotion regulation strategies and psychological symptoms among adults in Saudi Arabia. Using structural equation modeling, the findings revealed that mindfulness was positively associated with cognitive reappraisal but unrelated to expressive suppression. In turn, cognitive reappraisal predicted lower levels of depression and anxiety, whereas expressive suppression predicted higher psychological distress. Interestingly, mindfulness demonstrated small but significant direct positive associations with both depression and anxiety when emotion regulation variables were included, suggesting possible suppression effects. Furthermore, mediation analyses indicated that emotion regulation partially mediated the relationship between mindfulness and psychological symptoms, although total effects were non-significant.

These results align partially—but not fully—with prior international findings. Consistent with numerous studies, higher mindfulness predicted greater cognitive reappraisal and reduced reliance on expressive suppression, supporting the theoretical view that mindfulness enhances emotional awareness and flexible regulation (Gross, 2015; Garland et al., 2009). Similarly, the observed inverse associations between cognitive reappraisal and both depression and anxiety mirror prior evidence indicating that reappraisal serves as a protective factor against emotional distress (Aldao et al., 2010; Hu et al., 2014). The positive associations between expressive suppression and psychological symptoms are likewise congruent with the literature emphasizing the maladaptive nature of suppression, particularly in collectivistic contexts where emotional restraint may be socially reinforced but psychologically costly (Butler et al., 2007).

However, the small positive direct pathways observed between mindfulness and both depression and anxiety diverge from the bulk of prior research, which typically reports inverse associations (Brown et al., 2007; Keng et al., 2011). A plausible account is that, in a predominantly non-meditating community sample (54.2% had never practiced in our sample), dispositional mindfulness may reflect heightened monitoring of internal experience more than an acceptance-based stance. This distinction is central to Monitor and Acceptance Theory (MAT), which proposes that attention monitoring can increase the salience and intensity of unpleasant thoughts and sensations, whereas acceptance is the component expected to attenuate reactivity and protect wellbeing (Lindsay and Creswell, 2017).

In this context, greater self-awareness may initially make distress more noticeable and reportable before regulatory skills are sufficiently consolidated. This interpretation is also consistent with evidence that mindfulness-related practices can, for some individuals and under some conditions, be accompanied by challenging or distressing experiences (e.g., heightened emotional exposure, unpleasant arousal, or distressing cognitions), particularly when engagement is irregular or lacks adequate scaffolding (Lomas et al., 2015; Lindahl et al., 2017; Britton, 2019).

Importantly, this pattern may be further influenced by the Observe facet of mindfulness, which primarily reflects heightened attention to internal and external experiences. In the present study, mindfulness was modeled as a global construct, and the Observe facet was not examined separately. Prior research suggests that, particularly in non-meditating or clinical samples, higher observing may be associated with increased symptom reporting when not accompanied by acceptance or non-reactivity skills (Baer et al., 2006; Baer et al., 2008; Desrosiers et al., 2013).

Emerging evidence indicates that the Observe facet may function differently in clinical contexts. Psychometric studies have shown variability in the structure and behavior of this facet in clinical samples (Curtiss and Klemanski, 2014; Williams et al., 2014). More recently, anxiety sensitivity has been shown to differentially influence responses to Observe items in clinical populations, potentially inflating associations with distress-related outcomes (Moskow Diamond et al., 2025). Within this context, the positive direct associations observed in the current model may partly reflect heightened perceptual sensitivity to internal states rather than maladaptive effects of mindfulness per se.

Cultural norms surrounding emotional expression may further shape these associations. In Saudi society, emotional restraint is often socially valued, and expressive suppression may carry meanings related to respect, modesty, and self-control. While suppression is typically associated with maladaptive outcomes in Western samples (Gross and John, 2003), evidence from collectivist contexts suggests a more nuanced pattern. Studies from East Asian cultures, such as China and Japan, indicate that suppression is less consistently linked to psychopathology and may retain some social functionality (Matsumoto et al., 2008; Weiss et al., 2022). Within this cultural framework, the modest effects of suppression observed in the current model—and the indirect pathways linking mindfulness, regulation strategies, and distress—may reflect a hybrid pattern in which suppression remains socially adaptive in certain contexts but contributes to internal distress when employed rigidly or excessively.

The mediation results further emphasize the dual role of mindfulness. While mindfulness indirectly reduced psychological symptoms through enhanced reappraisal, its total effects were non-significant, suggesting that mindfulness operates through both protective and risk-related mechanisms depending on individual differences in regulation capacity. This aligns with emerging evidence that mindfulness without concurrent regulatory skills may increase emotional exposure without promoting resilience (Britton, 2019). In Saudi samples, where structured mindfulness programs are still scarce and often adapted from Western prototypes, this highlights the need for culturally tailored interventions that integrate mindfulness with cognitive and emotion-focused strategies to maximize psychological benefits.

The findings also have important implications for mental health practice and research in Saudi Arabia. Given that only a minority of participants practiced mindfulness regularly, promoting structured mindfulness-based interventions within educational and occupational settings could foster healthier emotional processing. However, these interventions must be adapted to respect cultural norms around privacy, modesty, and spirituality. Integrating mindfulness with Islamic contemplative traditions—such as reflective prayer (tafakkur) and remembrance (dhikr)—may enhance cultural acceptance and deepen emotional engagement, as suggested by recent culturally sensitive mindfulness frameworks in Arab contexts (Aldbyani, 2025; Kadir, 2025).

In summary, this study supports a nuanced model in which mindfulness contributes to emotional wellbeing primarily through its influence on cognitive reappraisal, while its direct links to distress may reflect early or incomplete stages of mindful awareness within a society still developing familiarity with such practices. These findings underscore the importance of cultural context in interpreting psychological pathways and call for more longitudinal and intervention-based research in non-Western settings.

## Conclusion

This study enhances understanding of how mindfulness contributes to emotional wellbeing within a non-Western context. The findings indicate that mindfulness is associated with more adaptive emotion regulation, particularly through greater use of cognitive reappraisal, which in turn relates to lower levels of depressive and anxious symptoms. At the same time, the small positive direct associations observed between mindfulness and psychological distress suggest that the effects of mindfulness may depend on the depth of practice, individual regulation capacity, and broader cultural familiarity with mindfulness-related skills.

Several considerations should be kept in mind when interpreting these findings. The cross-sectional design limits causal conclusions regarding the directionality of the observed relationships. In addition, all constructs were assessed using self-report measures, which may be influenced by shared method variance or response biases. Data were also collected anonymously through an online survey, which restricted the ability to conduct individual follow-up or obtain more detailed clinical information beyond self-reported history. Future research would benefit from longitudinal or intervention-based designs, the inclusion of behavioral or physiological indicators, and more comprehensive clinical assessments to further clarify these mechanisms.

Despite these considerations, the present findings underscore the importance of cultural context in interpreting mindfulness-related processes. In societies where mindfulness practices are still emerging and often adapted from Western models, mindfulness may initially heighten awareness of internal experiences before regulatory skills are fully consolidated. Accordingly, culturally sensitive approaches that integrate mindfulness with cognitive and emotion-focused strategies—and that resonate with local values and contemplative traditions—may be particularly important for maximizing psychological benefits. Continued research in non-Western settings is essential to refine theory, guide culturally grounded interventions, and advance a more globally inclusive understanding of mindfulness and emotional health.

## Data Availability

The datasets generated and analyzed during the current study are not publicly available due to participant confidentiality but are available from the corresponding author on reasonable request. Requests to access these datasets should be directed to HA, Halfayez1@ksu.edu.sa.
